# Growth Performance, Carcass Quality, and Lipid Metabolism in Krškopolje Pigs and Modern Hybrid Pigs: Comparison of Genotypes and Evaluation of Dietary Protein Reduction

**DOI:** 10.3390/ani14223331

**Published:** 2024-11-19

**Authors:** Martin Škrlep, Klavdija Poklukar, Milka Vrecl, Jana Brankovič, Marjeta Čandek-Potokar

**Affiliations:** 1 Agricultural Institute of Slovenia, Hacquetova ulica 17, SI-1000 Ljubljana, Slovenia; martin.skrlep@kis.si (M.Š.); klavdija.poklukar@kis.si (K.P.); 2 Veterinary Faculty, Institute of Preclinical Sciences, University of Ljubljana, Gerbičeva 60, SI-1000 Ljubljana, Slovenia; milka.vrecl@vf.uni-lj.si (M.V.); jana.brankovic@vf.uni-lj.si (J.B.); 3 Faculty of Agriculture and Life Sciences, University of Maribor, Pivola 10, SI-2311 Hoče, Slovenia

**Keywords:** Krškopolje pig, breed comparison, protein reduction, performance, carcass quality, lipid metabolism, meat quality

## Abstract

This study compared the performance, meat quality and adipose tissue characteristics of Krškopolje pigs and modern hybrid pigs under identical rearing conditions, besides examining the effects of dietary protein reduction in both genotypes. A total of 29 pigs (14 Krškopolje and 15 hybrids) were assigned to litter into two dietary groups (high and low protein). The low-protein diet for hybrid pigs corresponded to the high-protein diet for Krškopolje pigs. All diets were iso-energetic. Dietary protein reduction decreased growth rate and muscle development in modern hybrids but had no significant impact on performance, quality or metabolic traits in Krškopolje pigs. Genotype differences revealed that Krškopolje pigs had lower growth rates, less lean and more fat deposition, as reflected in thicker subcutaneous and higher intramuscular fat compared to modern hybrids. Krškopolje pigs also exhibited higher myoglobin concentration and fatty acid saturation. Lipogenic enzyme activity and histo-morphological traits behaved in a tissue-specific manner but still indicated a greater lipogenic potential in Krškopolje pigs. This study provides valuable insights into breed-specific responses to dietary changes and highlights the unique characteristics of Krškopolje pigs.

## Simple Summary

The Krškopolje pig has a reputation for being more resilient and having a lower protein requirement compared to modern breeds, which is important for sustainable agriculture. However, this difference needs to be proven by comparing the genotypes under the same conditions. The present study compares this indigenous Slovenian breed with modern hybrid pigs, investigating the effects of reduced dietary protein intake on growth and meat quality. Our research showed that dietary protein reduction in modern pigs led to a slight decrease in performance but not in Krškopolje pigs, confirming its lower protein requirement and better adaptability. Overall, the modern pigs grew faster and deposited more muscle, while the Krškopolje pigs deposited more fat. Their fat was also more saturated. The meat from Krškopolje pigs showed a better aptitude for processing into high-quality meat products. The results obtained can help farmers in rearing Krškopolje pigs more efficiently and thus preserve the breed and offer high-quality meat products.

## 1. Introduction

The Krškopolje pig, an indigenous Slovenian breed, is traditionally fattened to heavy weight. This breed has an important role in sustainable agricultural practices and the conservation of local genetic resources [1]. Local breeds such as the Krškopolje pig, which have not been genetically improved, often exhibit unique traits in terms of meat quality, higher fat and lower lean content, which are consistent with their lower protein requirements [2,3,4]. This characteristic is believed to increase resilience to food shortages and protein deficiency [5,6,7].

Reducing the protein content in pig diet is crucial for several reasons. Numerous studies on modern breeds have investigated the economic benefits and improved sustainability of protein reduction, as well as its effects on carcass and meat quality [8,9,10]. Feeding strategies restricting feed and/or protein intake are important in systems that aim to fatten pigs to a heavy weight for high-quality meat products [11,12]. Studies on local breeds such as the Iberian pig [13,14,15] and Cinta Senese [16,17] have shown that a reduction in protein content while meeting specific requirements, such as lysine and energy balance, does not significantly affect growth performance, nutrient utilization or meat quality and can improve traits such as intramuscular fat content and fatty acid composition [18]. Additionally, local breeds possess unique metabolic characteristics, including hormonal responses and fasting resistance, making them particularly interesting for further study [13,15,19].

In recent decades, the population of the Krškopolje pig breed has grown, increasing its importance to pig breeders and making it a focal point of scientific research. Previous studies [20,21,22,23] have investigated various aspects of Krškopolje pig breed: performances, carcass and meat quality, and biochemical and metabolic traits related to muscle and adipose tissue. These studies were conducted on pigs raised in various rearing conditions (i.e., intensive, extensive free-range, organic), whereas a comprehensive study comparing the Krškopolje pig with modern, lean-type pigs under identical conditions has so far not been performed. A preliminary assessment of the nutritional requirements of Krškopolje pigs has been reported [4]; however, more research is needed to understand the effects of protein deficiency/reduction at the physiological and biochemical level [23,24]

The aim of this study was to compare the response to protein reduction of fatty-type Krškopolje pigs and modern lean hybrid pigs reared under identical conditions and fattened to heavy weights. This involved characterizing Krškopolje pig on several aspects, including growth pattern, carcass and meat quality, as well as some biochemical, histomorphological and physiological properties, with particular attention to fat deposition and quality.

## 2. Materials and Methods

### 2.1. Animals Trial

The experiment included male surgical castrates of the Krškopolje pig breed and modern lean crossbred pigs (progeny of Landrace × Large White sows and Pietrain boars). The animals were allocated within breed and litter to two dietary treatments (low and high protein) at approximately 27 kg live weight, i.e., MH (modern breed, high protein diet, *n* = 7), MM (modern breed, medium protein diet, *n* = 7), KM (Krškopolje pig breed, medium protein diet, *n* = 7) and KL (Krškopolje pig breed, low protein diet, *n* = 8). Pigs were group-housed in four identical pens of 17.7 m^2^ indoor and 17.2 m^2^ outdoor area, full concrete flour, partly slatted (one-third of the surface), each pen equipped with automatic feeding and weighing systems (Compident MLP II PRO, Schauer Agrotronic GmbH, Prambachkirchen, Austria) allowing individual feed intake dosing and recording. A three-phase feeding plan was applied using five feed mixtures (Table 1). Due to the assumed breed-specific requirements, different crude protein (CP) levels were tested in modern and Krškopolje pig breeds. Feed composition is presented in Table 1, with CP content differing roughly by 2 percentage points between feed mixtures. The experiment aimed at fattening to heavy weights (>160 kg) targeting app. 750 g/day in modern hybrids. Ad libitum feed allowance was planned [25] until week 12, thereafter limited to 2.5 kg/day/pig until week 20 and then to 3.0 kg/day/pig until slaughter. All animals were given equal amount of feed (Table 2), which was possible using automatic feeding stations with individual control of intake.

The chemical composition of feed mixtures (dry matter, crude fat, protein, fiber and ash), as well as its amino acid and chemical composition, were analyzed according to standard procedures [26]. The live weight of the animals was recorded daily using scales integrated into the automatic feeding system, whereas *Longissimus lumborum* muscle (LL) and backfat thickness measurements were taken at the end of each feeding phase. The measurements were taken at the level of the last rib by applying an ultrasound device (Draminski 4Vet Mini, Draminski S.A., Olsztyn, Poland). The values obtained were used to calculate average daily gain (ADG), in addition to muscle and backfat gains during individual feeding periods and during the entire trial.

### 2.2. Modeling of Nutritional Requirements

The information on growth performance and feed mixtures was transferred to InraPorc*^®^* (version 1.8.1.0., INRA Saint-Gilles, France)a software tool, enabling the evaluation of the performance and nutrient utilization or requirements [27] to evaluate the extent to which the tested diets met the pigs’ nutritional needs. Calculation of nutrient requirements requires creating animal profiles that describe the potential for growth and feed intake of the studied pigs. In the model, daily feed intake in MJ of net energy was modeled as a gamma function of body weight, expressing daily feed intake in multiples of net energy intake for maintenance. Animal growth was described as protein deposition modeled by a Gompertz function. In the next step, the InraPorc*^®^* simulation was conducted to assess whether the two diets applied in each breed (high and low protein content) met the specific protein and amino acid requirements for the growth performance of each genotype (see Supplementary Figures S1–S4).

### 2.3. Slaughter, Carcass and Meat Quality Evaluation and Sampling

After being fattened for 178 days in total, the pigs were transported to a commercial abattoir (transport duration of 1 h at a distance of 80 km) and slaughtered in one slaughter batch according to the routine procedure with CO_2_ stunning, followed by bleeding, vapor scalding, dehairing and evisceration. Carcasses were then split in half and weighed. The thickness of the backfat at the levels of withers, last rib and over the gluteus medius muscle (GM) were measured in addition to the measurement of muscle thickness (defined as the shortest distance between the dorsal edge of the vertebral canal and the cranial edge of the GM muscle) at the carcass split line, and lean meat percentage calculated according to the method approved for Slovenia [28]. Carcass length (defined as the distance between the cranial part of the pubic bone and the cranial part of the first cervical vertebra) was also measured.

At 45 min after the slaughter, the pH value was measured in LL (using Testo 205 pH meter, Testo SE & Co, KGaA, Lenzkirch, Germany) at the level of the last rib. At the same location, the samples of backfat (inner layer), LL, semispinalis capitis muscle (SSC) and liver (lobus caudatus) were taken for biochemical analyses. Samples were immediately frozen in liquid nitrogen and stored at −80 °C for subsequent analyses. The samples of backfat (both layers, 2–3 cm^3^) were taken and stored in 10% buffered formalin to be later subjected to histological analyses.

After the pig carcasses were cooled overnight at 0–2 °C to reach internal muscle temperature under 7 °C, they were cut at the level of the last rib, perpendicularly to the spine. On the freshly cut surface, marbling and subjective color evaluation (Japanese scale 1–6), in addition to objective color measurement with a Minolta chromameter (CIE L*, a*, b*), LL cross-sectional area and the area of overlying subcutaneous fat measurement was undertaken (as described by Batorek et al. [29]). Subsequently, two LL portions were cut, trimmed of adjacent fat and connective tissues and used for determination of shear force, water holding capacity and chemical composition. For drip loss measurements, cylindrical samples of approximately 10 g were excised, weighted and stored for 24 or 48 h at 4 °C when they were reweighed. For evaluating thawing and cooking loss, LL muscle samples (8 × 5 × 4 cm) were weighed, packed in vacuum bags and frozen at −20 °C. After thawing, samples were reweighed and subjected to thermal treatment until they reached an internal temperature of 72 °C, being subsequently cooled, weighed again and used for shear force determination. For this measurement, three individual cores were excised using a half-inch cylindrical knife and then cut perpendicularly to the muscle fiber direction on a texture analyzer (TA Plus, Ametek Lloyd Instruments Ltd., Fareham, UK) applying 60° V-shaped knife with rectangular edges. Another sample of LL was frozen in liquid nitrogen and homogenized to a fine dust to determine oxidation (carbonyl groups and thiobarbituric reactive substances—TBARS), collagen, fatty acid composition and myoglobin (cf.5.2).

### 2.4. Histological Analyses

After being fixed in formalin solution, backfat samples were dehydrated and embedded in paraffin. Tissue sections of 5 µm (microtome SMR2000R, Leica Nusloch, Germany) were then cut in a vertical plane through both external and internal backfat layers and stained with hematoxylin and eosin (HE) and cover slipped using Gemini AS slide stainer and ClearVue cover slipper (Thermo Fischer Scientific, Winsford, UK). For histomorphometric analysis, images were taken with a light microscope (Eclipse Ni.U, Nikon, Tokyo, Japan) equipped with a digital camera (DS-Fi1, Nikon, Tokyo, Japan). Three images per sample were acquired at 10× objective magnification, and the number of adipocytes and adipocyte cross-sectional area (CSA) were determined. These parameters were measured separately in the inner and outer backfat layers. The adipocyte number was counted manually within 3 regions of interest (ROI; each 1.1 mm^2^ in size) per sample. The CSA value of adipocytes was determined in 3 ROIs using the protocol of Fiji 1.54f software [30], as shown in Supplementary Figure S5 and described below. The HE-stained images of external and internal backfat layers were converted to 8-bit images (Supplementary Figure S5B), which were then subjected to the following processing steps: noise correction with a Gaussian blur filter (sigma = 2.00); a default threshold of 189 was used for segmentation. Post-processing of the images included mask adjustment (erosion and opening) and inversion to identify the adipocytes as objects and the connective tissue between the adipocytes as background (Supplementary Figure S5C). Only the adipocytes falling inside the range of specified parameters (size 400–13,000 µm², cells on the edges were excluded) were examined (Supplementary Figure S5D). On average, 149 adipocytes were analyzed per ROI. Each image was manually checked for segmentation and corrected when necessary.

### 2.5. Biochemical Analyses

#### 2.5.1. Muscle Chemical Composition

To determine muscle chemical composition, minced LL and SSC samples were scanned by a near-infrared spectroscopy device (NIR Systems 6500 Monochromator, FOSS NIR System, Silver Spring, MD, USA). The spectra obtained were then utilized for the prediction of protein, moisture and intramuscular fat (IMF) content using internal calibrations previously developed at the Agricultural Institute of Slovenia.

#### 2.5.2. Protein and Lipid Oxidation

The concentration of thiobarbituric acid reactive substances (TBARS) was used to assess the level of lipid oxidation. The method of Lynch and Frei [31] was used. Samples were homogenized in 0.15 M KCl buffer with the addition of 0.1 mM butylated hydroxytoluene to prevent further oxidation. The obtained homogenate was centrifuged, and the supernatant was incubated with 50 mM NaOH, 1% 2-thiobarbituric, and 2.8% trichloroacetic acids (both *w*/*v*) for 10 min at 100 °C to enable the development of pink chromogen. After cooling, the chromogen was extracted by adding *n*-butanol, and its absorbance was measured at 535 nm (using BioSpectrometer Fluorescence, Eppendorf, Wesseling-Berzdorf, Germany), enabling the calculation of TBARS.

To determine the level of protein oxidation, myofibril isolates were prepared, and protein carbonyl groups were determined according to the method described by Rezar et al. [32]. The isolates were divided into four aliquots for each sample, two of them treated with 2M HCl and the other two with 2M HCl and 2,4-dinitrophenylhydrazine (DNPH, 0.2%, *w*/*v*). All samples were then added 50% trichloroacetic acid, and the emerging precipitate was centrifuged. The resulting pellets were washed three times with ethyl acetate in ethanol to remove residual DNPH and subsequently dissolved in guanidine HCl (6 M) in phosphate buffer (2 mM). The HCl-treated samples were utilized to measure protein concentration (calculated from the absorbance measured at 280 nm), while the concentration of carbonyl groups was measured in DNPH-treated samples at 370 nm and expressed as nmol/mg proteins. Absorbances were measured using BioSpectrometer Fluorescence (Eppendorf, Wesseling-Berzdorf, Germany)

#### 2.5.3. Myoglobin

Muscle myoglobin content was determined in LL according to the description provided based on the method introduced by Trout [33]. Briefly, LL muscle (2 g) was homogenized in 0.04 M potassium phosphate buffer (20 mL, pH 6.5). After filtration, the homogenate (4 mL) was mixed with 10% Triton X–100 (1.4 mL) and 0.065 M sodium nitrate (0.1 mL) solutions. Following stirring and incubation (22 °C, 60 min), the absorbance was measured at 409 and 307 nm using BioSpectrometer Fluorescence (Eppendorf GmbH, Wesseling-Berzdorf, Germany), and it was used for the calculation of myoglobin concentration.

#### 2.5.4. Collagen

For the determination of total collagen, the procedure described by ISO 3496 [34] was used. The procedure is based on the spectrophotometric determination of hydroxyproline multiplied by factor 8 to calculate collagen content. In short, homogenized LL samples were thermally treated (90 min at 77 °C) and subjected to hydrolyzation in sulfuric acid (16 h at 105 °C). The hydrolyzate was then filtered and incubated in the solution of propan-2-ol, perchloric acid, chloramine-T and p-dimethylaminobenzaldehyde to develop a color reaction. Hydroxyproline content was determined by measuring absorbance at 558 nm (Biospectrometer Fluorescence, Eppendorf GmbH, Wesseling-Berzdorf, Germany). For insoluble collagen, samples were first heated in 25% Ringer’s solution (90 min at 77 °C) and centrifuged. The supernatant was discarded, and the pellet was then analyzed, as in the case of total collagen. Soluble collagen was calculated as the difference between total and insoluble fractions.

#### 2.5.5. Fatty Acid Composition

Fatty acid composition of feed mixtures (Supplementary Table S1), subcutaneous fat and LL muscle was conducted as reported previously by Škrlep et al. [35]. After finely grinding the samples in liquid nitrogen (app. 0.5 g of LL muscle and feed mixtures and 0.1 g of subcutaneous fat tissue), they were transmethylated in situ, as described by Park and Goins [36]. This process involved a 0.5M NaOH solution of dichloromethane and methanol (50 min at 90 °C), followed by the addition of a methanol solution of 14% BF_3_ (10 min at 90 °C). This resulted in the formation of fatty acid methyl esters (FAME) that were extracted with hexane and separated by gas chromatography (Hewlett Packard 6890, Agilent, Santa Clara, CA, USA) equipped with SP-2560 GC column (100 m × 0.25 mm i.d. × 0.20 µm, Supelco, Bellefonte, PA, USA) with nitrogen carrier gas and flame ionization detector (FID). The starting temperature was set at 80 °C, being increased as follows: to 160 °C at a rate of 20 °C, to 198 °C at a rate of 1 °C/min and to 250 °C at a rate of 1 °C/min, the total run time being 94.4 min. The injector and FID temperatures were set at 220 and 300 °C, respectively. Separate FAMEs were identified using a mixture of standards (Supelco 37 Component FAME mix) and nonadecanoic acid (C19:0, Sigma Aldrich, St. Louis, MO, USA) as an internal standard for determining concentrations.

#### 2.5.6. Lipogenic Enzymes

Lipogenic enzyme activities, including glucose 6-phosphate dehydrogenase (G6PDH), malic enzyme (ME), citrate cleavage enzyme (CCL) and fatty acid synthase (FAS) were determined in muscle (LL and SSC), subcutaneous fat and liver, following the protocol of Bazin and Ferré [37]. Samples of tissue (1 g) were finely ground in liquid nitrogen and homogenized in sucrose buffer (0.25 M sucrose, 1 mM dithiothreitol, 1 mM ethylenedinitrilotetraacetic acid and 1 mM phenylmethylsulfonyl fluoride). After centrifugation, enzyme activities were assessed by measuring absorbance at 340 nm against the blank and expressed as nmol of oxidized NADH/NADPH or reduced NADP per minute per gram of tissue. In some of the examined tissues, lipogenic enzyme activity (CCL in LL, FAS in LL and SSC) could not be detected.

### 2.6. Statistical Analysis

A one-way ANOVA was performed using the GLM procedure of SAS statistical software, version 9.4 (SAS Institute Inc., Cary, NC, USA), and least square means were compared (using the ESTIMATE option) to test the effect of breed (i.e., MM vs. KM) or dietary treatment within the breed (i.e., HM vs. MM and KM vs. KL). A *p*-value < 0.05 was considered statistically significant, while *p* < 0.10 was considered a trend.

## 3. Results

### 3.1. Nutritional Needs and Growth Performance

Modeling results for modern crosses in the MH group (Supplementary Figures S1 and S2) showed no amino acid deficiencies for the average animal. Some minor deficiencies in methionine, tryptophan and threonine could be noted at the population level occurring after the transition to the lowest protein diet (12.6% CP). The animals in the MM group showed a deficit in several amino acids. These were tryptophan, threonine, isoleucine and valine (considering the average animal) and additionally methionine and histidine (at population level). The deficiencies occurred again at the transition to the lowest protein diet (10.4% CP) and in the case of tryptophan and threonine already at the first cut of protein level (12.6% CP). In the Krškopolje pig (Supplementary Figures S3 and S4), modeling results showed no deficits in essential amino acids for an average animal in both groups, KM and KL. A small deficit of threonine was noted, but only at the population level.

Regarding the effect of nutrition on growth parameters (Table 3) it influenced a few traits in the modern crossbreed. Pigs on a reduced protein diet (MM) had lower (*p* < 0.05) LL thickness at the end of the second and third experimental periods (i.e., before slaughter), which was reflected in lower LL gain during the second (*p* < 0.05) experimental period, a trend towards lower LL gain during the third (*p* < 0.10) experimental period and also in lower (*p* < 0.05) total LL gain. Regarding growth rate, lower ADG (11%, *p* < 0.05) was observed for MM than MH pigs in the last experimental phase. In the Krškopolje breed, the differences in diet had no significant impact on the measured growth parameters.

The differences between breeds reared under the same rearing conditions (i.e., MM vs. KM) were more pronounced. Starting from almost the same initial weight, Krškopolje pigs (i.e., the KM group) had lower weight than modern crossbred pigs (MM group) at the end of all experimental phases, resulting in a 13% difference in weight at the end of the fattening trial and overall, 15% lower ADG. Accordingly, a trend towards smaller (*p* < 0.1) thickness of LL muscle (during all experimental phases and overall) was observed in KM than in MM, together with lower LL gain (during the second fattening phase and overall, *p* < 0.05). On the other hand, Krškopolje pigs showed significantly higher lipid deposition. Through the entire fattening process, KM pigs had greater backfat gain than MM pigs, which was reflected in 2.3-fold thicker backfat at the end of the trial.

### 3.2. Carcass and Meat Quality Traits

In modern crosses, reducing the feed protein content affected muscle development (Table 4). Namely, we observed smaller (*p* < 0.05) muscle thickness at the GM level and smaller (*p* < 0.001) LL cross-sectional area in MM than in MH animals. As for the effects of protein reduction in the Krškopolje pig, the differences were only noted for meat color. The KM group had higher (*p* < 0.05) values for the instrumental color parameters a* and b* compared to the KL group.

Breed differences (i.e., comparing MM vs. KM) were, however, more pronounced; KM had lower carcass weight (*p* < 0.01) and shorter carcasses (*p* < 0.05), smaller muscle thickness at GM and 1.7-fold smaller LL cross-sectional area (*p* < 0.001) than MM. As expected, higher carcass fatness was noted for KM than MM pigs. This was expressed in a greater thickness of subcutaneous fat (1.3 to 2-fold difference, *p* < 0.001), regardless of the location of measurement. Several differences in meat quality were also found between MM and KM. Krškopolje pigs had higher pH24 (*p* < 0.05) and more intensive meat color (both subjectively assessed and instrumentally measured; see also Supplementary Figure S6).

### 3.3. Chemical Composition

Diet (i.e., protein level) effect in modern crosses was noted only for collagen being slightly (*p* < 0.10) more soluble in MH than MM, while in Krškopolje pig, higher myoglobin concentration (*p* < 0.05), higher *n*-3 PUFA and lower *n*-6/*n*-3 PUFA ratio was found in KM than KL group (Table 5, Supplementary Table S2).

The comparison between breeds (MM vs. KM) showed higher IMF content in KM than MM, both in LL (2.6-fold, *p* < 0.001) and SSC muscles (1.7-fold, *p* < 0.01). Consequently, moisture content was lower in KM than in MM (*p* < 0.05 in LL and *p* < 0.01 in SSC). In addition to its higher IMF content, the IMF of Krškopolje pigs had a higher content of SFA (*p* < 0.001) and MUFA (*p* < 0.01) and a lower content of PUFA (*p* < 0.001), including both *n*-6 (*p* < 0.01) and *n*-3 (*p* < 0.05) PUFA, but with a lower *n*-6/*n*-3 PUFA ratio (*p* < 0.001). A similar result was observed for subcutaneous fat, although the differences were less pronounced (Table 5, Supplementary Table S3). A higher level of LL muscle lipid oxidation (i.e., TBARS) was observed in KM compared to MM (*p* < 0.001). A higher concentration of myoglobin (*p* < 0.001), primarily responsible for meat color, was also observed in KM.

### 3.4. Lipogenic Enzyme Activity

Protein reduction did not affect the activity of lipogenic enzymes in modern crosses or Krškopolje pigs, the only exception being CCL activity in the SSC muscle (higher in KL than in KM, *p* < 0.05; Table 6). On the other hand, some breed-related differences were observed, and these were quite tissue-specific. In subcutaneous fat, G6PD activity tended to be lower (*p* < 0.10), whereas the activities of the enzymes CCL and FAS were higher (*p* < 0.001) in KM than in MM. The difference in the latter is particularly marked as Krškopolje pigs had a 3-fold higher FAS activity than modern hybrids. While no differences were observed in LL, a tendency of higher ME activity (*p* < 0.10) and higher G6PD activity (*p* < 0.05) was observed in the SSC muscle of KM than in MM. In the liver, ME enzyme activity tended to be higher (*p* < 0.10), and G6PD enzyme activity was lower (*p* < 0.05) in KM than in pigs from the MM group.

### 3.5. Histo-Morphological Traits

Reducing the protein content in the feed had no major effect on modern crosses or Krškopolje pigs (Table 7; Supplementary Figure S7). The only difference was a slightly (*p* < 0.10) higher number of adipocytes per ROI in MM than in KM in the outer layer of backfat tissue. As for breed differences, a trend toward a larger area (*p* < 0.10) and a lower number of adipocytes per ROI (*p* < 0.05) was observed in KM than in MM in the inner layer of backfat.

## 4. Discussion

### 4.1. Nutritional Needs and Growth Performance

In modern crossbred pigs, around 2 percentage point reduction in CP was reflected in a deficit of some essential amino acids, resulting in reduced muscle deposition and ADG. This result agrees with general knowledge regarding the effects of low-protein diets on swine [9]. In contrast, the same (i.e., medium) protein level, as well as further reduction of dietary protein level in Krškopolje pig, provided more than the required amounts of all amino acids for the local breed. Due to the lack of experimental data, the exact information for the Krškopolje pig was not available. Despite the fact that we have set the experimental CP levels relatively low, the requirements limit was not reached to test the response of the Krškopolje breed to protein deficiency. As to the literature reports, protein requirements reported for different local pig breeds are generally low; for example, Sirtori et al. [16,38] and Aquilani et al. [17] found that 12% CP in the diet is sufficient for the Cinta Senese breed in the growing phase (28–65 kg), while on average for the entire growing and fattening period, 10% CP is adequate. Still, the differences among individual local breeds can be quite substantial. According to Brossard et al. [4], the Krškopolje pig had the highest protein deposition among nine local breeds examined in their study (with 65% higher values than the above-mentioned Cinta Senese breed). It is, therefore, a bit surprising that no deficits were found even for pigs on a low-protein diet (KL group), especially as the animals were not fed ad libitum in the last fattening period. Nevertheless, it should be emphasized that the study of Brossard et al. [4] was conducted within a limited weight range (40–100 kg) and in different environmental conditions. Additionally, lower requirements can also be explained by the fact that protein reduction enhances production efficiency through digestive efficiency [39] or gastrointestinal health [9].

Few comparisons between local and modern pig breeds in identical environmental conditions can be found in the literature, here comprised of the Krškopolje pig. This is the first study that directly compares Krškopolje pigs and modern hybrids in the same conditions (feeding, management) of intensive indoor systems. The comparison of breeds is, however, to be interpreted with caution. Due to the different dietary protein requirements (MM being deficient and KM in excess), the physiological response can also be influenced. Still, the observed differences, such as lower growth rate, muscle deposition and higher fat deposition, are in line with the literature comparing local, non-selected breeds to modern lean breeds. As pointed out by Barea et al. [40], this may be either related to the lower nutrient retention ability linked to less efficient small intestine structural properties (as noted in their study on Iberian vs. Landrace × Large White pigs) or to the differences in tissue protein and energy utilization. In general, many studies show that local breeds exhibit lower growth rates compared to modern breeds, though breed variation can be considerable [41,42]. Consistent with the present findings, several other local breeds, such as Basque [43], Iberian [40,44], Creole [45] and Cinta Senese [46], also demonstrate lower growth rates and higher fat deposition compared to modern breeds. Regarding the Krškopolje pig, the only other comparative study [23] was conducted in free-range conditions, where modern crosses showed a lower growth rate attributed to poorer adaptability to harsh environments.

### 4.2. Carcass, Meat Quality and Chemical Composition

Reduction of dietary protein affected carcass muscularity of modern hybrids but not Krškopolje pigs, which is consistent with the results on muscle growth. The lack of differences in meat quality and composition, as well as muscle and subcutaneous fat biochemical parameters, reflects the relatively small effects of protein reduction on growth and carcass traits. The only exception was a slightly increased amount of insoluble collagen in MM compared to MH. This could be related to slightly slower growth, which has been shown to correlate negatively with collagen solubility as more cross-linking occurs between collagen molecules [47]. Obviously, the differences were not large enough to noticeably affect meat quality characteristics (namely shear force [48], which differs only numerically). The effect of dietary protein level on muscle pigment in the Krškopolje breed (higher myoglobin content in the KL group) is consistent with the effect on instrumental color, namely the parameter a * (redness). Regarding the effect of protein restriction on color or pigment, the literature is not consistent. While Wang et al. [49] reported a decrease in the value of the parameter a *, several studies [50,51,52] observed no differences in instrumental color or myoglobin [53], while others found an increase in color parameter values [54,55].

The greater amount of intramuscular fat observed in the Krškopolje breed compared to modern hybrids in the present study is consistent with a generally higher fat content of the carcass [3,56] and is already a well-established trait in local pig breeds [24,57]. In this study, however, this was confirmed under identical conditions of feeding and management. According to the conclusions of Zhao et al. [58], the reasons for the higher IMF in fatty pigs are their higher capacity for lipogenesis and fatty acid transport, in addition to the lower capacity of lipolysis. The observed higher saturation of fatty acids in Krškopolje pigs can also be explained by higher carcass adiposity [56]. As to the fatty acid composition, the scientific literature on local breeds generally shows higher levels of MUFA and lower levels of PUFA compared to modern hybrid pigs, while reports on SFA vary widely [24]. In agreement with the present study, a preliminary comparison of Krškopolje and modern pigs [23] showed the greatest differences in PUFA and MUFA but less in SFA. According to the literature [56,59], the reasons for the characteristic fatty acid profile are mainly greater de novo synthesis (SFA) and greater desaturation capacity (MUFA), which leads to a dilution reduction of PUFA being a fraction that cannot be synthesized by the pigs themselves and is obtained exclusively from the feed. In the present study, the breed-related differences in fatty acid composition are much more pronounced in IMF than in subcutaneous fat. The former is subject to greater and more rapid changes and is also more strongly affected by various influences, including genetic and metabolic factors [56,59,60]. With regard to the fatty acid profile, the lower *n*-6/*n*-3 ratio observed in pigs from Krškopolje is also interesting. Although a lower ratio could be considered beneficial for health, it should be emphasized that the values observed in the present study are still very high (above 20), while the recommended ratio is below 4 [61]. The higher myoglobin content of Krškopolje pigs compared to modern crossbreds is consistent with the more oxidative muscle profile of local breeds [62,63] and agrees with a higher meat color score (i.e., more intense color) and redness parameter a * [64]. The higher degree of intramuscular fat oxidation (i.e., TBARS) in Krškopolje pigs does not relate to its fatty acid composition but may be related to a higher amount of muscle myoglobin [65,66] or a generally higher IMF content [67,68], both factors being positively associated with oxidation. It should also be noted that the TBARS level was much lower than that associated with sensory perceived rancidity (i.e., 0.5 mg/kg, [69,70] and also had no negative effects on meat quality in our study (e.g., on shear force and water holding capacity, [71,72,73]. The increased TBARS value can nevertheless be associated with a slightly more yellowish color of LL of Krškopolje pigs. Namely, the parameter b* is positively associated with oxidative changes [68,74].

### 4.3. Lipogenic Enzyme Activities

In modern crosses, the lack of dietary protein level effect on lipogenic enzymes reflects the lack of differences in adipose tissue deposition. In the Krškopolje pig, higher activity of one of the enzymes (i.e., CCL in SSC muscle) indicates slightly higher lipogenesis associated with a low-protein diet. The literature on the effect of reducing dietary protein is not very consistent. The reason may be in the specifics of the individual studies (different genotypes, experimental design or type and extent of protein reduction, tissues studied, etc.). Some studies on Chinese breeds [75,76] suggest an increase in lipogenic activity, others [15,77] find no effects on lipogenic enzyme activity or find tissue-dependent effects [13]. Similar to our study, Tejeda et al. [15] conclude that protein reduction in the local Iberico breed did not significantly affect the activity of lipogenic enzymes or the fatness characteristics of the carcass.

In terms of lipogenic enzyme activity, the breed-related differences were much more pronounced but tissue-dependent. A similar situation has been described in various studies in pigs [15,52,60] and suggests different regulatory mechanisms in fat metabolism in different tissues such as subcutaneous fat, IMF or liver [13,78,79,80]. In this study, enzyme activities were generally higher in the Krškopolje than in the modern cross, which contradicts our previous study [23] showing higher activities in modern crossbreds, but the study was conducted in different conditions (free-range rearing) and using a different crossbreed (Duroc crosses). The scientific literature reports variable results and points to the importance of physiological maturity (which is associated with lower activity) but nevertheless concludes that the activity of lipogenic enzymes is generally higher in local breeds (in line with higher fatness reflecting a greater ability to store lipids [24]). This has been demonstrated, for example, for the enzymes G6PDH, ME and FAS in Alentejana and Iberian vs. Large White in subcutaneous [13,81] and also in IMF in the Iberico, Wujin, Basque and Meishan breeds [13,58,82].

### 4.4. Histo-Morphological Traits of Adipose Tissue

No effect of dietary protein in modern lean or local fatty pigs on the size of adipocytes found in the present study is consistent with the absence of effects on fat deposition. Despite observing certain deficiencies in amino acids, the imbalances were apparently too small to shift energy utilization towards adiposity. In accordance with our findings, the literature also confirms that the size of adipocytes in porcine adipose tissues is greater in fatty than lean breeds [79,83,84]. In general, adiposity (the size of fat depots) is positively correlated with the size of adipocytes, while the increase in fat depots is mostly due to cell hypertrophy and only to a lesser extent to hyperplasia [85,86,87,88]. This association was also confirmed in the present study, as we observed a 30% larger adipocyte cross-sectional area in Krškopolje pigs than in modern crosses, which is approximately consistent with a 40% larger thickness of backfat measured at the level of the last rib. The mentioned differences, however, refer exclusively to the inner subcutaneous fat layer, while they were much smaller in the outer layer. It is already known from older research that in pigs, the lipogenic activity of the inner layer of subcutaneous fat is greater than in the outer layer [85,89,90,91]. Similar to our case, Alfonso et al. [82] showed a greater contribution of the inner layer of subcutaneous fat in creating differences between the local Basque and modern Large White.

## 5. Conclusions

A reduction in dietary protein by approximately 2 percentage points in growing and fattening production phases resulted in some amino acid deficiencies in the fattening phase but did not greatly impact the overall performance of modern lean crossbred pigs or local fat-type pigs. However, the effects were more pronounced in lean than local breeds, confirming lower protein requirements of the less-performing genotype. The absence of dietary effect in Krškopolje pigs combined with modeled nutrient requirements also indicated that the protein threshold for this breed had not been reached and has the potential to be lowered further.

These two divergent genotypes, usually raised in different environments, were compared in identical feeding and management conditions to evaluate more precisely the differences in performances. Overall, the results confirmed the characteristics of local fat-type pigs, i.e., slower growth and lower feed efficiency but higher lipogenic potential and advantage in terms of meat quality (intramuscular fat). In conclusion, the results highlight the importance of adapting feeding strategies to suit the nutritional requirements of local breeds, especially in niche product production where meat quality is a key focus. Sufficiently optimizing (i.e., lowering) feed protein content could enhance the cost-effectiveness and sustainability of feeding strategies for local pig breeds.

## Figures and Tables

**Table 1:** Ingredients and nutritional composition of experimental feeds.

Item	Feed 1	Feed 2	Feed 3	Feed 4	Feed 5
Ingredient (%)					
Maize	31.08	23.93	30.00	32.00	26.35
Barley	23.00	32.00	40.00	45.00	55.76
Wheat	18.00	20.00	10.00	10.00	9.81
Wheat feed flour	.	.	5.00	4.88	.
Soybean meal	17.82	10.52	5.39	2.90	.
Sunflower meal	1.50	2.00	2.60	.	1.20
Rapeseed meal	3.73	7.26	2.00	.	.
Soybean oil	0.22	.	0.38	0.25	0.45
Molasses	1.00	1.00	1.00	1.00	1.00
Calcium carbonate	1.05	1.04	1.04	1.07	1.01
Monocalcium phosphate	0.97	0.52	0.60	0.70	0.81
Sodium chloride	0.50	0.50	0.48	0.48	0.48
Magnesium oxide	0.20	0.10	0.10	0.10	0.10
L-lysine-HCl	0.19	0.23	0.44	0.60	0.37
DL-methionine	.	.	0.01	0.07	0.08
L-threonine	.	.	0.06	0.06	.
L-tryptophan	.	.	0.01	0.01	.
Zeolite	.	.	.	.	1.50
Vitamins and trace mineral mixture	0.75	0.90	0.89	0.89	0.89
Chemical composition (%)					
Dry matter	87.2	87.5	88.2	88.1	88.6
Crude protein	16.7	14.6	12.6	10.4	9.3
Crude fat	2.4	2.4	2.7	2.6	2.4
Crude fiber	3.6	4.1	3.9	3.5	2.9
Ash	5.4	4.5	5.4	4.5	5.1
Nutritional values					
Metabolic energy ^1^, MJ/kg	12.8	12.7	12.8	12.9	12.6
Net energy ^1^, MJ/kg	9.45	9.53	9.64	9.91	9.73
Lysine, g/kg	9.27	8.61	8.38	8.12	6.15
Methionine, g/kg	2.70	2.60	2.27	2.47	2.40
Cystine, g/kg	3.17	3.13	2.58	2.23	2.14
Tryptophan, g/kg	1.90	1.77	1.42	1.18	1.03
Threonine, g/kg	6.07	5.56	4.95	4.03	3.21
Phenylalanine, g/kg	7.83	7.03	5.72	4.65	4.44
Tyrosine, g/kg	5.38	4.77	3.74	2.99	2.88
Leucine, g/kg	12.88	11.48	9.65	8.08	7.48
Isoleucine, g/kg	6.70	5.99	4.62	3.55	3.34
Valine, g/kg	7.91	7.35	5.96	4.89	4.65
Histidine, g/kg	4.18	3.77	3.01	2.39	2.19
Arginine, g/kg	10.11	8.90	6.77	5.03	4.53

^1^ Calculated by InraPorc^®^, Version 1.8.1.0, INRA, Saint-Gilles, France.

**Table 2:** Experimental design regarding the duration of individual feeding phases and diet.

Trait	Phase 1	Phase 2	Phase 3
Duration, days	56	59	63
Feed intake, kg/pig/day	1.64	2.42	2.81
Treatment group			
MH	Feed 1	Feed 2	Feed 3
MM	Feed 1	Feed 3	Feed 4
KM	Feed 1	Feed 3	Feed 4
KL	Feed 1	Feed 4	Feed 5

MH = modern hybrid pigs fed high protein diet; MM = modern hybrid pigs fed medium protein diet; KM = Krškopolje pigs fed medium protein diet; KL = Krškopolje pigs fed low protein diet.

**Table 3:** Growth performance traits of modern hybrid pigs and Krškopolje pigs fed diets differing in crude protein content.

					Protein Level Difference		Breed Difference		
Trait ^1^	MH	MM	KM	KL	MH-MM (*p*-Value)	KM-KL (*p*-Value)	MM-KM (*p*-Value)	RMSE	Significance (*p*-Value)
Weight 1, kg	27.8	27.0	26.8	27.1	0.6686	0.8836	0.9017	3.640	0.9485
Weight 2, kg	66.4	66.6	59.1	61.0	0.9500	0.5371	0.0262	5.905	0.0542
Weight 3, kg	114.5	111.0	95.8	97.5	0.4638	0.7070	0.0032	8.729	0.0005
Weight 4, kg	164.5	155.3	135.6	136.7	0.1111	0.8903	0.0015	10.365	<0.0001
BFT 1, mm	4.0	4.2	7.4	7.8	0.7349	0.4593	<0.0001	1.171	<0.0001
BFT 2, mm	5.9	5.7	12.5	12.1	0.8416	0.6922	<0.0001	1.959	<0.0001
BFT 3, mm	10.3	10.0	22.3	23.4	0.8306	1.4306	<0.0001	2.720	<0.0001
BFT 4, mm	15.2	14.2	32.6	32.3	0.6387	0.8486	<0.0001	3.936	<0.0001
LLT 1, mm	26.1	25.2	20.6	18.9	0.4664	1.1751	0.0011	2.348	<0.0001
LLT 2, mm	38.9	38.2	30.6	31.5	0.7223	0.6079	0.0006	3.451	<0.0001
LLT 3, mm	52.3	48.5	36.9	38.2	0.0307	0.4055	<0.0001	3.115	<0.0001
LLT 4, mm	61.2	54.2	42.6	44.1	0.0024	0.4666	<0.0001	3.873	<0.0001
BFG 1–2, mm/day	0.03	0.03	0.11	0.08	0.7068	0.1698	0.0020	0.0404	0.0053
BFG 2–3, mm/day	0.07	0.07	0.17	0.19	0.7195	0.1465	<0.0001	0.0327	<0.0001
BFG 3–4, mm/day	0.08	0.07	0.16	0.14	0.6403	0.2958	0.0003	0.0429	0.0004
BFG 1–4, mm/day	0.06	0.06	0.14	0.14	0.5808	0.6684	<0.0001	0.0230	<0.0001
LLG 1–2, mm/day	0.23	0.23	0.18	0.23	0.9339	0.2232	0.2089	0.0725	0.5035
LLG 2–3, mm/day	0.23	0.17	0.11	0.11	0.0298	0.7759	0.0378	0.0484	0.0002
LLG 3–4, mm/day	0.14	0.09	0.09	0.09	0.0760	0.9487	0.9710	0.0510	0.2037
LLG 1–4, mm/day	0.20	0.16	0.12	0.14	0.0179	0.1858	0.0080	0.0252	<0.0001
ADG 1–2, g/day	687.6	706.0	577.4	606.4	0.6494	0.4623	0.0036	74.926	0.0082
ADG 2–3, g/day	815.4	753.7	621.2	618.0	0.1587	0.9389	0.0045	79.398	<0.0001
ADG 3–4, g/day	793.8	703.1	632.9	617.4	0.0079	0.6140	0.0343	58.682	<0.0001
ADG 1–4, g/day	767.5	720.8	611.5	614.2	0.1457	0.9313	0.0017	58.123	<0.0001

^1^ Numerials next to the traits from 1 to 4 represent individual time points during the experiment (i.e., 1—at the start of the experiment; 2—at the end of the first phase, within which all the animals were fed the same feed, 56 days from the start of the experiment; 3—at the end of the second phase, within which the first change in the diet took place, 115 days from the start of the experiment; 4—at the end of the third phase, within which the second change in the diet took place, 178 days from the start of the experiment, followed by slaughter). MH = modern hybrid pigs fed high protein diet; MM = modern hybrid pigs fed medium protein diet; KM = Krškopolje pigs fed medium protein diet; KL = Krškopolje pigs fed low protein diet; RMSE = root mean square error of the model; BFT = backfat thickness; LLT = *Longissimus lumborum* muscle thickness; BFG = backfat gain; LLG = *Longissimus lumborum* muscle gain; ADG = average daily gain.

**Table 4:** Carcass and meat (*Longissimus lumborum*) quality traits of modern hybrid pigs and Krškopolje pigs fed diets differing in crude protein content.

					Protein Level Difference		Breed Difference		
Trait	MH	MM	KM	KL	MH-MM (*p*-Value)	KM-KL (*p*-Value)	MM-KM (*p*-Value)	RMSE	Significance (*p*-Value)
Carcass weight, kg	133.0	125.5	108.9	109.3	0.1356	0.926	0.0022	9.11	<0.0001
Dressing, %	80.9	80.8	80.2	80.1	0.9180	0.8525	0.5149	1.59	0.7076
Muscle GM, mm	83.4	77.6	66.1	70.1	0.0352	0.1304	0.0002	4.92	<0.0001
BFT GM, mm	19.9	18.6	37.0	39.3	0.6622	0.4318	0.0001	5.44	<0.0001
BFT withers, mm	40.4	41.8	55.8	57.0	0.6478	0.6790	0.0001	5.77	<0.0001
BFT last rib, mm	31.3	27.9	38.7	38.5	0.2118	0.9415	0.0004	4.90	0.0004
LEA, cm^2^	76.8	63.9	38.3	37.8	0.0002	0.7747	<0.0001	5.63	<0.0001
LEA fat, cm^2^	25.1	22.2	40.3	39.2	0.4543	0.8704	<0.0001	7.13	<0.0001
CL, cm	91.3	90.7	87.0	88.5	0.6911	0.2862	0.0150	2.66	0.0197
pH45	6.16	6.04	6.14	6.37	0.4202	0.1049	0.5219	0.267	0.1375
pH24	5.36	5.40	5.47	5.53	0.2606	0.1105	0.0212	0.059	<0.0001
Color score, 1–6	2.4	2.5	3.8	3.5	0.9105	0.2579	0.0002	0.58	0.0001
Marbling score, 1–7	1.3	1.4	3.1	2.7	0.8767	0.4318	0.0009	0.85	0.0005
L*	56.5	54.8	54.4	53.7	0.2060	0.6101	0.7414	2.42	0.1869
a*	7.3	7.5	10.4	8.4	0.7845	0.0231	0.0022	1.60	0.0042
b*	7.2	7.0	8.2	7.2	0.6872	0.0385	0.0193	0.87	0.0752
Drip loss 24h, %	7.3	6.9	5.4	5.4	0.7481	0.9752	0.2346	2.30	0.2905
Drip loss 48h, %	9.4	9.0	7.5	7.8	0.7406	0.8123	0.2411	2.31	0.3582
Thawing loss, %	11.9	11.4	11.2	11.4	0.6787	0.8361	0.8401	2.36	0.9367
Cooking loss, %	29.4	29.1	28.7	28.8	0.7968	0.9055	0.7315	2.16	0.9311
Shear force, *n*	52.9	55.9	52.0	49.3	0.4835	0.5087	0.3635	7.85	0.4563

MH = modern hybrid pigs fed high protein diet; MM = modern hybrid pigs fed medium protein diet; KM = Krškopolje pigs fed medium protein diet; KL = Krškopolje pigs fed low protein diet; RMSE = root mean square error of the model; Muscle GM = the shortest distance between the dorsal edge of the vertebral canal and cranial edge of *Gluteus medius* muscle at the carcass split line; BFT GM = backfat thickness above the *Gluteus medius* muscle; LEA = *Longissimus lumborum* cross-sectional area; LEA fat = area of subcutaneous fat corresponding to *Longissimus lumborum* cross-sectional area; CL = carcass length measured from cranial edge of first rib to cranial edge of the pubic bone symphisis; L*, a*, b* = instrumental color parameters denoting lightness, redness and yellowness, respectively.

**Table 5:** Biochemical traits of *Longissimus lumborum*, *Semispinalis capitis* and backfat in modern hybrid pigs and Krškopolje pigs fed diets differing in crude protein content.

					Protein Level Difference		Breed Difference		
Trait	MH	MM	KM	KL	MH-MM (*p*-Value)	KM-KL (*p*-Value)	MM-KM (*p*-Value)	RMSE	Significance (*p*-Value)
*Longissimus lumborum* muscle									
IMF, %	1.0	1.4	3.6	4.1	0.3294	0.3038	0.0001	0.91	<0.0001
Proteins, %	23.8	23.7	23.5	23.3	0.5085	0.3990	0.3385	1.5	0.0811
Moisture, %	73.7	73.3	71.9	71.8	0.4721	0.8928	0.0114	0.99	0.0013
SFA, g/100 g fatty acids	37.90	37.84	42.40	41.47	0.9572	0.3764	0.0002	2.0005	0.0002
MUFA, g/100 g fatty acids	37.80	39.70	45.49	47.20	0.3276	0.3649	0.0055	3.5658	<0.0001
PUFA, g/100 g fatty acids	24.31	22.46	12.10	11.33	0.4769	0.7579	0.0004	4.779	<0.0001
Myoglobin, mg/g	1.21	1.31	1.84	1.58	0.3975	0.0186	<0.0001	0.202	<0.0001
TBARS, raw, µg MDA/kg	0.44	0.45	0.58	0.56	0.8003	0.4845	0.0004	0.063	0.0001
Carbonyl raw, nmol/mg protein	1.03	1.11	1.15	1.18	0.1231	0.6135	0.4170	0.097	0.0355
Collagen, total, mg/g	0.41	0.46	0.43	0.47	0.2310	0.3296	0.5570	0.072	0.3974
Collagen solubility, %	24.4	17.4	14.0	12.9	0.0767	0.7691	0.3705	7.086	0.0207
*Semispinalis capitis* muscle									
IMF, %	10.5	10.8	17.9	17.2	0.9109	0.7285	0.0020	3.82	0.0011
Proteins, %	20.1	19.4	19.0	18.8	0.2887	0.6895	0.5827	1.15	0.2284
Moisture, %	68.8	68.4	62.7	63.3	0.8237	0.6950	0.0026	1.19	0.0013
Subcutaneous fat									
SFA, g/100 g fatty acids	43.62	43.22	45.59	46.71	0.7045	0.2780	0.0328	1.9627	0.0059
MUFA, g/100 g fatty acids	41.49	41.20	43.45	42.92	0.6805	0.4414	0.0035	1.3039	0.0077
PUFA, g/100 g fatty acids	14.89	15.59	10.97	10.36	0.3537	0.4034	<0.0001	1.3711	<0.0001

MH = modern hybrid pigs fed high protein diet; MM = modern hybrid pigs fed medium protein diet; KM = Krškopolje pigs fed medium protein diet; KL = Krškopolje pigs fed low protein diet; RMSE = root mean square error of the model; SFA = saturated fatty acids; MUFA = monounsaturated fatty acids; PUFA = polyunsaturated fatty acids; TBARS = thiobarbituric acid reactive substances; MDA = malondialdehyde; IMF = intramuscular fat.

**Table 6:** Lipogenic enzyme activities in modern hybrid pigs and Krškopolje pigs fed diets differing in crude protein content. Activities are expressed in nmol of oxidized/reduced NADP and NADH/NAD per minute per gram of wet tissue.

					Protein Level Difference		Breed Difference		
Enzymes	MH	MM	KM	KL	MH-MM (*p*-Value)	KM-KL (*p*-Value)	MM-KM (*p*-Value)	RMSE	Significance (*p*-Value)
Subcutaneous fat									
ME	1132.5	1011.9	992.3	1080.7	0.2726	0.4038	0.8570	201.15	0.5473
G6PD	1019.7	993.0	885.2	907.8	0.6457	0.6892	0.0728	107.60	0.0739
CCL	172.3	145.9	283.2	311.5	0.4677	0.4198	0.0007	66.82	<0.0001
FAS	15.4	13.5	40.0	35.7	0.7840	0.4956	0.0004	12.19	0.0004
*Longissimus lumborum* muscle									
ME	219.1	225.7	271.8	292.1	0.8339	0.5066	0.1501	58.10	0.0633
G6PD	25.7	27.0	30.8	29.9	0.8894	0.9279	0.6984	17.85	0.9425
*Semispinalis capitis* muscle									
ME	334.0	291.6	370.3	414.1	0.3575	0.3286	0.0944	84.76	0.0595
G6PD	99.0	100.6	161.9	171.3	0.9467	0.6952	0.0187	45.60	0.0058
CCL	77.2	54.5	41.6	96.3	0.3945	0.0412	0.6287	49.13	0.1696
Liver									
ME	197.3	163.8	217.0	226.9	0.2903	0.7438	0.0983	57.97	0.1971
G6PD	2330.4	2534.1	2106.0	2166.7	0.2121	0.6969	0.0125	297.6	0.0520
CCL	12.3	8.8	13.5	16.1	0.4481	0.6204	0.3700	165.27	0.4771
FAS	24.2	18.3	26.9	21.9	0.4032	0.4698	0.2223	12.37	0.6416

MH = modern hybrid pigs fed high protein diet; MM = modern hybrid pigs fed medium protein diet; KM = Krškopolje pigs fed medium protein diet; KL = Krškopolje pigs fed low protein diet; RMSE = root mean square error of the model; ME = malic enzyme; G6PD = glucose-6-phosphate dehydrogenase; CCL = citrate cleavage enzyme; FAS = fatty acid synthase.

**Table 7:** Backfat histomorphological traits in modern hybrid pigs and Krškopolje pigs fed diets differing in crude protein content.

					Protein Level Difference		Breed Difference		
Trait	MH	MM	KM	KL	MH-MM (*p*-Value)	KM-KL (*p*-Value)	MM-KM (*p*-Value)	RMSE	Significance (*p*-Value)
Outer backfat layer									
ADP cross-sectional area (µm^2^)	2938.3	3338.9	3748.8	3658.2	0.3693	0.8324	0.3583	819.57	0.2581
									0.0580
Number of ADP (per ROI)	223.1	191.7	182.6	167.6	0.0824	0.3827	0.6033	32.50	0.0222
Inner backfat layer									
ADP cross-sectional area (µm^2^)	3429.7	3526.4	4574.4	4814.3	0.8545	0.6390	0.0555	976.10	0.0208
									0.0108
Number of ADP (per ROI)	184.7	186.1	142.3	138.3	0.9315	0.8021	0.0133	30.79	0.0060

ADP= adipocyte; ROI = region of interest, corresponding to the size of 1.1 mm^2^; MH = modern hybrid pigs fed high protein diet; MM = modern hybrid pigs fed medium protein diet; KM = Krškopolje pigs fed medium protein diet; KL = Krškopolje pigs fed low protein diet; RMSE = root mean square error of the model.

## Data Availability

The data presented in this study are available upon direct request to the authors.

## Supplementary Materials

The following supporting information can be downloaded at https://www.mdpi.com/article/10.3390/ani14223331/s1: Supplementary Table S1: Fatty acid composition of feed mixtures. Supplementary Table S2: Fatty acid composition of *Longissimus lumborum* muscle in modern hybrid pigs and Krškopolje pigs fed diets differing in crude protein content. Supplementary Table S3: Fatty acid composition of backfat in modern hybrid pigs and Krškopolje pigs fed diets differing in crude protein content. Supplementary Figure S1: Amino acid (lysine, methionine, tryptophan, threonine) requirements and supply according to the diet in modern crossbreed (MH—modern hybrid pigs, high protein diet; MM—modern hybrid pigs, reduced protein diet). Supplementary Figure S2: Amino acid (isoleucine, valine, histidine) requirements and supply according to the diet in modern crossbreed (MH—modern hybrid pigs, high protein diet; MM—modern hybrid pigs, medium protein diet). Supplementary Figure S3: Amino acid (lysine, methionine, tryptophan, threonine) requirements and supply according to the diet in Krškopolje breed (KM—Krškopolje pig, medium protein diet; KL—Krškopolje breed, low protein diet). Supplementary Figure S4: Amino acid (isoleucine, valine, histidine) requirements and supply according to the diet in Krškopolje breed (KM—Krškopolje pig, medium protein diet; KL—Krškopolje breed, low protein diet). Supplementary Figure S5: Determination of the cross-sectional area of adipocytes (CSA) in external and internal backfat layers. Images were subjected to the macro protocol in Fiji 1.54f software. A representative photomicrograph of an ROI of backfat tissue stained with HE (2A) was converted to an 8-bit image (2B), segmented (threshold of 189) and converted to a binary image that was refined and inverted (2C). The result of the macro protocol was labeled adipocytes (2D), whose CSA was automatically measured (all images 10× magnification). Supplementary Figure S6: Image of *Longissimus lumborum* cross-sections behind the level of last rib in modern hybrid (MP) and Krškopolje pig (KP). Supplementary Figure S7: Representative photomicrographs of the external and internal backfat layers of modern hybrid pigs and Krškopolje pigs fed diets with different crude protein content. MH = modern hybrid pigs fed high protein diet; MM = modern hybrid pigs fed medium protein diet; KM = Krškopolje pigs fed medium protein diet; KL = Krškopolje pigs fed low protein diet; EBL = external internal backfat layer; IBL = internal backfat layer; hematoxylin and eosin staining; scale bars = 500 µm.
