# Supplementary material for: Growth Performance, Carcass Quality, and Lipid Metabolism in Krškopolje Pigs and Modern Hybrid Pigs: Comparison of Genotypes and Evaluation of Dietary Protein Reduction
Source: Animals (Basel). 2024 Nov 19;14(22):3331. doi: 10.3390/ani14223331 (PMC11591021; doi:10.3390/ani14223331)
Supplement: Supplementary file 1 [file animals-14-03331-s001.zip › Supplementary Figure S1.pdf]

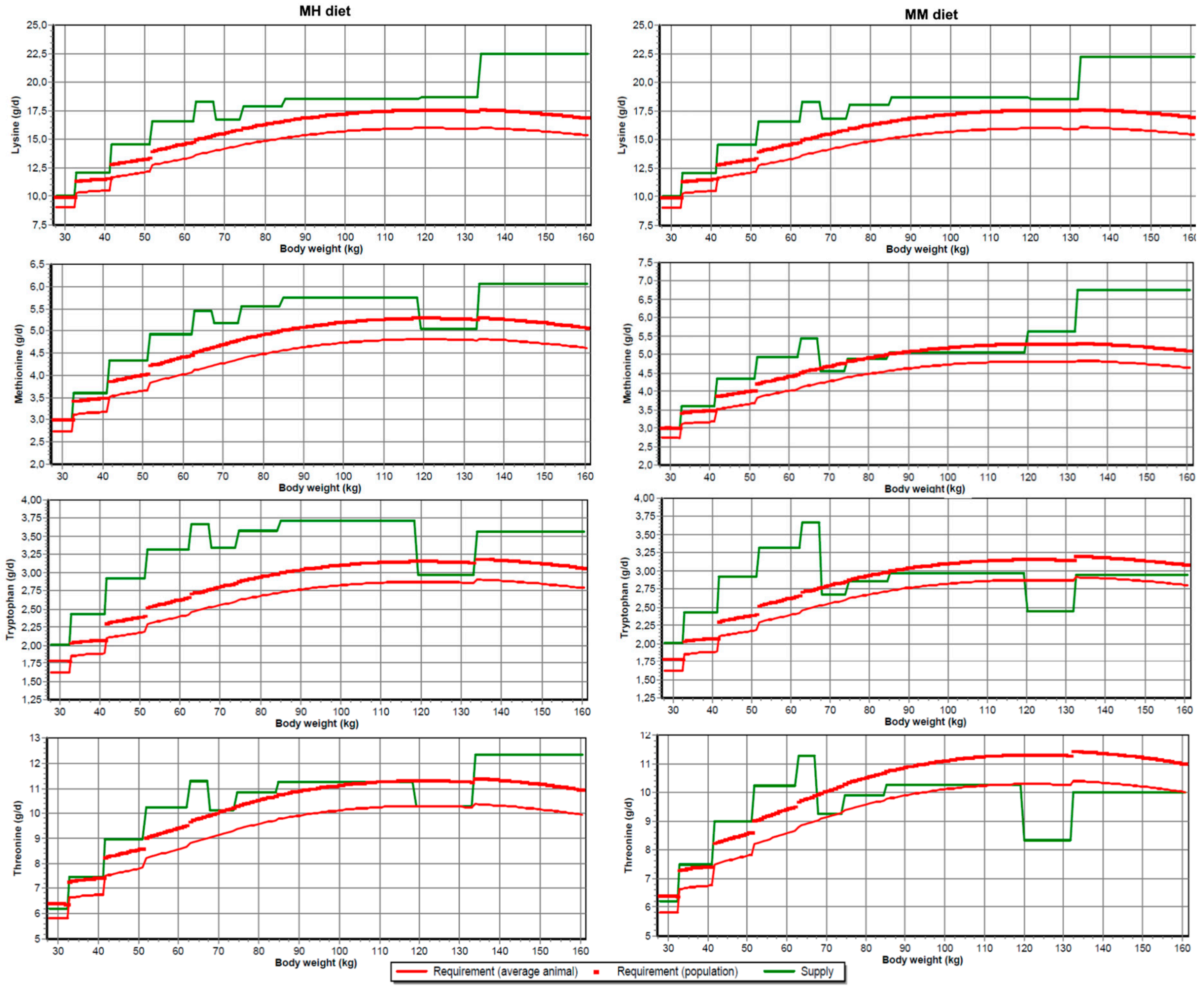

Supplementary Figure S1: Amino acid (lysine, methionine, tryptophan, threonine) requirements and supply according to the diet in modern hybrid pigs (MH – modern hybrid pigs, high protein diet; MM – modern hybrid pigs, reduced protein diet).
