# Supplementary material for: Growth Performance, Carcass Quality, and Lipid Metabolism in Krškopolje Pigs and Modern Hybrid Pigs: Comparison of Genotypes and Evaluation of Dietary Protein Reduction
Source: Animals (Basel). 2024 Nov 19;14(22):3331. doi: 10.3390/ani14223331 (PMC11591021; doi:10.3390/ani14223331)
Supplement: Supplementary file 1 [file animals-14-03331-s001.zip › Supplementary Figure S5.pdf]

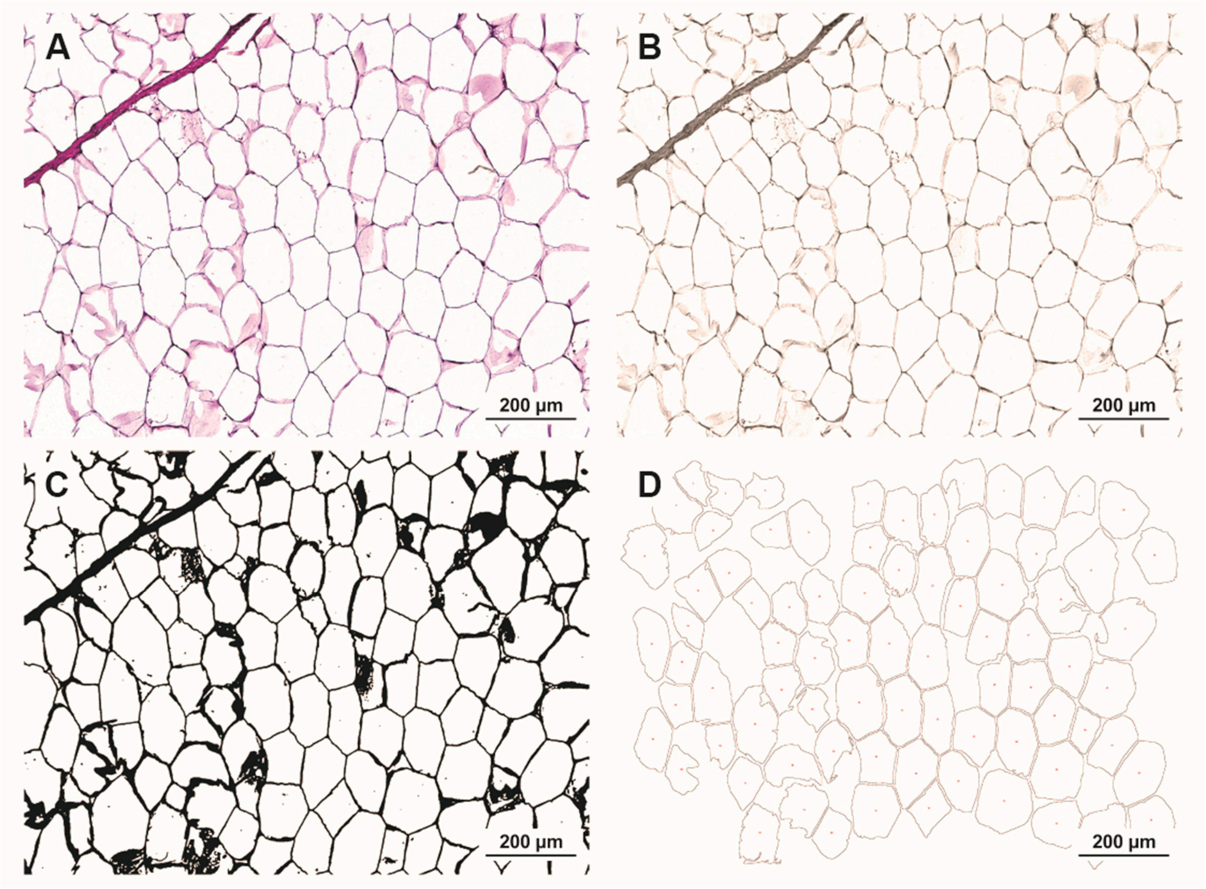

Supplementary Figure S5: Determination of the cross-sectional area of adipocytes (CSA) in external and internal backfat layers. Images were subjected to the macro protocol in Fiji 1.54f software, a representative photomicrograph of an ROI of backfat tissue stained with HE (2A) was converted to an 8-bit image (2B), segmented (threshold of 189) and converted to a binary image that was refined and inverted (2C). The result of the macro protocol was labeled adipocytes (2D) whose CSA was automatically measured (all images 10x magnification).
