# Supplementary material for: Growth Performance, Carcass Quality, and Lipid Metabolism in Krškopolje Pigs and Modern Hybrid Pigs: Comparison of Genotypes and Evaluation of Dietary Protein Reduction
Source: Animals (Basel). 2024 Nov 19;14(22):3331. doi: 10.3390/ani14223331 (PMC11591021; doi:10.3390/ani14223331)
Supplement: Supplementary file 1 [file animals-14-03331-s001.zip › Supplementary Figure S6.pdf]

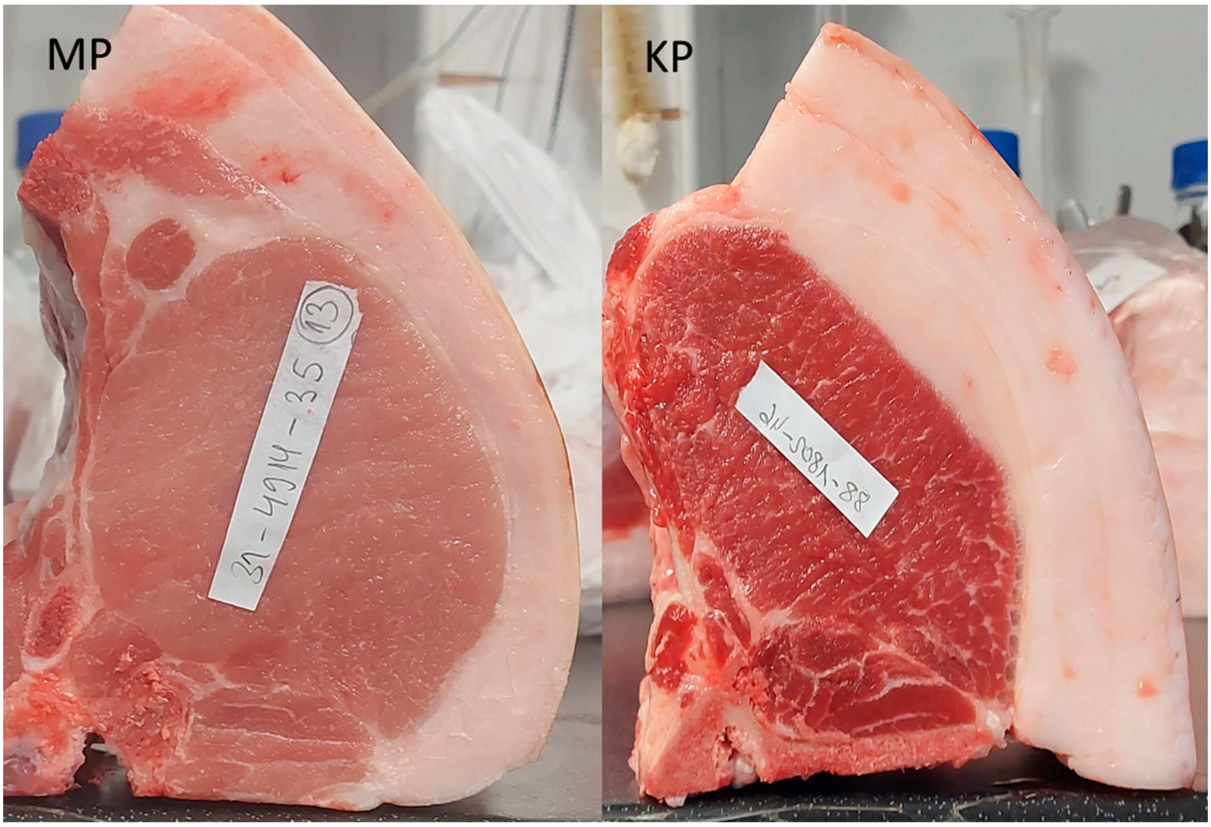

Supplementary Figure S6: Image of *longissimus lumborum* muscle cross sections behind the level of last rib in modern hybrid (MP) and Krškopolje pig (KP).
