# Supplementary material for: Growth Performance, Carcass Quality, and Lipid Metabolism in Krškopolje Pigs and Modern Hybrid Pigs: Comparison of Genotypes and Evaluation of Dietary Protein Reduction
Source: Animals (Basel). 2024 Nov 19;14(22):3331. doi: 10.3390/ani14223331 (PMC11591021; doi:10.3390/ani14223331)
Supplement: Supplementary file 1 [file animals-14-03331-s001.zip › Supplementary Figure S7.pdf]

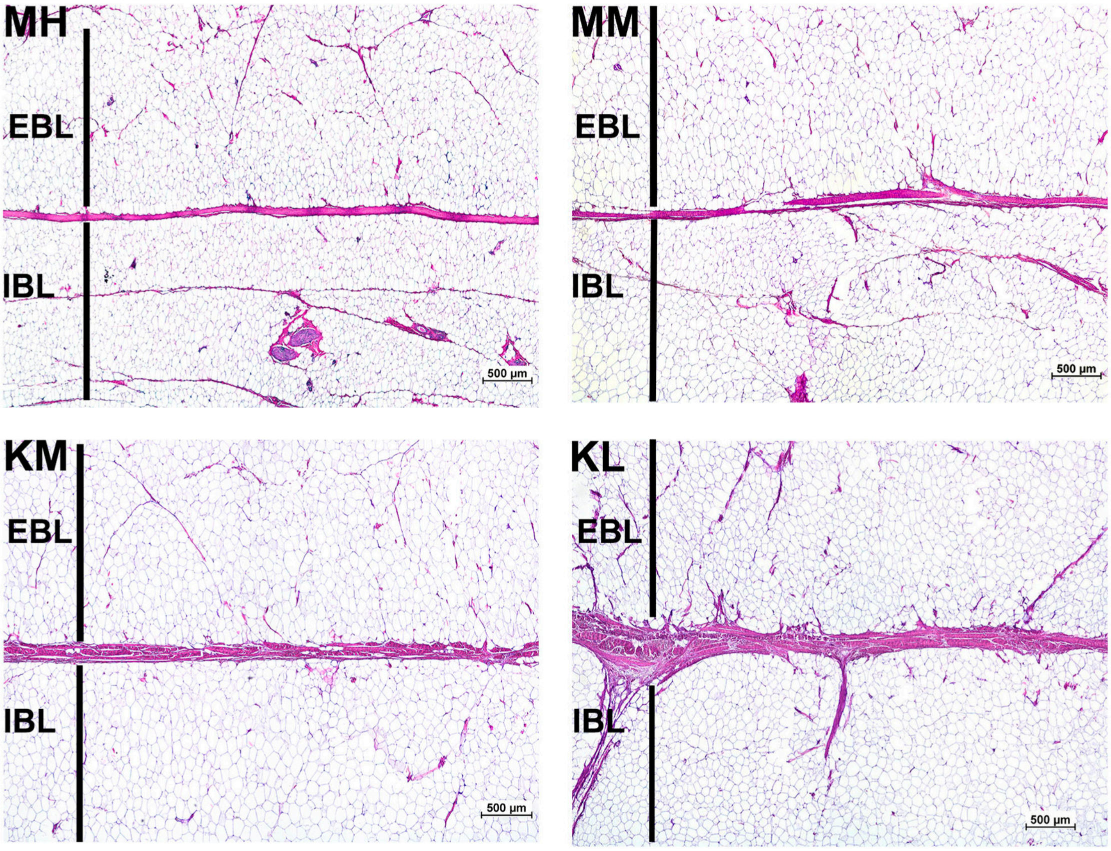

Supplementary Figure S7: Representative photomicrographs of the external and internal backfat layers of modern hybrid pigs and Krškopolje pigs fed diets with different crude protein content. MH = modern hybrid pigs fed high protein diet; MM = modern hybrid pigs fed medium protein diet; KM = Krškopolje pigs fed medium protein diet; KL = Krškopolje pigs fed low protein diet; EBL = outer backfat layer; IBL = inner backfat layer; hematoxylin and eosin staining; scale bars = 500 µm.
