# Supplementary material for: Growth Performance, Carcass Quality, and Lipid Metabolism in Krškopolje Pigs and Modern Hybrid Pigs: Comparison of Genotypes and Evaluation of Dietary Protein Reduction
Source: Animals (Basel). 2024 Nov 19;14(22):3331. doi: 10.3390/ani14223331 (PMC11591021; doi:10.3390/ani14223331)
Supplement: Supplementary file 1 [file animals-14-03331-s001.zip › Supplementary Table S1.pdf]

Supplementary Table S1. Fatty acid composition of feed mixtures. Results are presented in g per 100g fatty acids.

Trait	Feed 1	Feed 2	Feed 3	Feed 4	Feed 5
C14:0	0.16	0.21	0.18	0.16	0.16
C15:0	0.07	0.07	0.06	0.06	0.05
C16:0	16.97	16.09	15.51	16.01	16.00
C16:1 n-7	0.19	0.24	0.19	0.13	0.31
C17:0	0.06	0.06	0.05	0.05	0.05
C17:1 n-7	0.06	0.06	0.05	0.05	0.05
C18:0	2.79	2.68	2.58	2.05	2.02
C18:1 *trans* n-9	0.06	0.07	0.06	0.03	0.02
C18:1 *cis* n-9	22.75	22.76	21.61	20.99	21.32
C18:2 n-6	51.55	52.21	54.38	55.38	55.28
C20:0	0.38	0.33	0.35	0.34	0.35
C20:1 n-9	0.41	0.46	0.44	0.48	0.46
C18:3 n-3	3.55	3.92	3.75	3.47	3.38
C21:0	0.06	0.03	0.03	0.07	0.06
C20:2 n-6	0.13	0.08	0.07	0.07	0.06
C22:0	0.34	0.32	0.31	0.27	0.26
C22:1 n-9	0.06	0.05	0.05	0.06	0.06
C23:0	0.06	0.05	0.05	0.04	0.05
C20:5 n-3	0.22	0.09	0.18	0.18	0.17
C24:0	0.10	0.17	0.06	0.07	0.05
C24:1 n-9	0.04	0.05	0.04	0.05	0.05
C22:6 n-3	0.02	0.01	0.02	0.02	0.01
