# Supplementary material for: Growth Performance, Carcass Quality, and Lipid Metabolism in Krškopolje Pigs and Modern Hybrid Pigs: Comparison of Genotypes and Evaluation of Dietary Protein Reduction
Source: Animals (Basel). 2024 Nov 19;14(22):3331. doi: 10.3390/ani14223331 (PMC11591021; doi:10.3390/ani14223331)
Supplement: Supplementary file 1 [file animals-14-03331-s001.zip › Supplementary Table S2.pdf]

Supplementary Table S2. Fatty acid composition of *longissimus lumborum* muscle in modern hybrid pigs and Krškopolje pigs fed diets differing in crude protein content. Results are presented in g per 100 g fatty acids.

Trait	MH	MM	KM	KL	MH-MM	MM-KM	KM-KL	RMSE	P
C12:0	0.08	0.08	0.09	0.10	0.7574	0.0483	0.4378	0.0108	0.0150
C14:0	1.07	1.10	1.54	1.59	0.7827	0.0003	0.6211	0.1985	<0.0001
C16:0	23.13	23.27	26.33	23.27	0.8354	<0.0001	0.9526	1.1814	<0.0001
C14:1 n-5	.	.	0.04	0.04	.	.	0.6562	0.0080	0.6562
C15:0	.	0.06	0.03	0.04	.	<0.0001	0.0282	0.0034	0.0002
C16:1 n-7	3.06	3.13	4.24	4.33	0.7879	0.0003	0.7283	0.4903	<0.0001
C17:0	0.27	0.25	0.18	0.21	0.4645	<0.0001	0.0344	0.0277	<0.0001
C17:1 n-7	0.09	0.08	0.04	0.20	0.8200	0.5711	0.0169	0.1220	0.0847
C18:0	12.98	12.81	13.97	12.99	0.7726	0.0542	0.0896	1.0658	0.1911
C18:1 *cis* n-9	33.77	35.67	40.29	41.67	0.2834	0.0130	0.4187	3.2367	0.0002
C18:1 *trans* n-9	0.18	0.17	0.18	0.19	0.5003	0.7776	0.4343	0.0234	0.7152
C18:2 n-6	16.40	15.38	8.51	8.14	0.5346	0.0003	0.8203	3.0323	<0.0001
C18:3 n-6	0.20	0.16	0.07	0.06	0.1321	0.0010	0.6625	0.0434	<0.0001
C18:3 n-3	0.43	0.39	0.31	0.30	0.0141	<0.0001	0.6261	0.0314	<0.0001
C20:0	0.15	0.16	0.18	0.16	0.8501	0.1144	0.0943	0.0255	0.2210
C20:1 n-9	0.65	0.61	0.70	0.75	0.4662	0.0947	0.3608	0.0946	0.0591
C20:2 n-6	0.33	0.34	0.25	0.25	0.3503	<0.0001	0.8416	0.0354	<0.0001
C20:3 n-6	0.66	0.60	0.31	0.31	0.5391	0.0035	0.9793	0.1670	0.0004
C20:3 n-3	0.06	0.06	0.04	0.04	0.6275	0.0678	0.7132	0.0085	0.2003
C20:4 n-6	5.75	5.24	2.45	2.11	0.4952	0.0009	0.6382	1.3847	<0.0001
C20:5 n-3	0.22	0.18	0.08	0.07	0.1812	0.0035	0.7166	0.0561	<0.0001
C22:0	0.26	0.21	0.10	0.09	0.1210	0.0022	0.6515	0.0598	<0.0001
C22:1 n-9	0.10	0.10	0.03	0.03	0.8726	<0.0001	0.5452	0.0141	<0.0001
C22:6 n-3	0.32	0.20	0.09	0.08	0.0802	0.1512	0.8770	0.1218	0.0085
PUFA n-3	0.98	0.75	0.52	0.45	0.0193	0.0233	0.4856	0.1783	<0.0001
PUFA n-6	23.32	21.72	11.59	10.88	0.5214	0.0004	0.7706	4.6222	<0.0001
PUFA n-6/n-3 ratio	24.08	29.15	22.40	24.12	0.0045	0.0003	0.2842	3.0382	0.0021

MH = modern hybrid pigs fed high protein diet; MM = modern hybrid pigs fed medium protein diet; KM = Krškopolje pigs fed medium protein diet; KL = Krškopolje pigs fed low protein diet; RMSE = root mean square error of the model; PUFA = polyunsaturated fatty acids.
