# Supplementary material for: Growth Performance, Carcass Quality, and Lipid Metabolism in Krškopolje Pigs and Modern Hybrid Pigs: Comparison of Genotypes and Evaluation of Dietary Protein Reduction
Source: Animals (Basel). 2024 Nov 19;14(22):3331. doi: 10.3390/ani14223331 (PMC11591021; doi:10.3390/ani14223331)
Supplement: Supplementary file 1 [file animals-14-03331-s001.zip › Supplementary Table S3.pdf]

Supplementary Table S3. Fatty acid composition of backfat in modern hybrid pigs and Krškopolje pigs fed diets differing in crude protein content. Results are presented in g per 100 g fatty acids.

Trait	MH	MM	KM	KL	MH-MM (*p* – value)	MM-KM (*p* – value)	KM-KL (*p* – value)	RMSE	Significance (*p* – value)
C10:0	0.07	0.11	0.07	0.09	0.0735	0.0512	0.4370	0.0312	0.1889
C12:0	0.07	0.07	0.07	0.08	0.4049	0.3822	0.0429	0.0063	0.0317
C14:0	1.31	1.26	1.37	1.48	0.3632	0.0632	0.0446	0.1050	0.0024
C15:0	0.05	0.06	0.04	0.04	0.1461	0.0285	0.5386	0.0103	0.1108
C16:0	25.13	24.92	26.89	27.72	0.7453	0.0040	0.1794	1.1619	0.0001
C16:1 n-7	1.64	1.67	1.85	2.00	0.8027	0.2118	0.2741	0.2582	0.0424
C17:0	0.19	0.08	0.09	0.11	0.0233	0.9156	0.6515	0.0848	0.0800
C17:1 n-7	0.28	0.27	0.18	0.21	0.6139	0.0055	0.2071	0.0518	0.0053
C18:0	16.53	16.50	16.83	16.98	0.9684	0.6202	0.8034	1.2030	0.8394
C18:1 *trans* n-9	0.22	0.26	0.21	0.23	0.2593	0.1582	0.6398	0.0623	0.5081
C18:1 *cis* n-9	38.23	38.08	39.95	39.34	0.8112	0.0052	0.3074	1.1448	0.0133
C18:2 n-6	12.93	13.67	9.51	9.04	0.2764	<0.0001	0.4711	1.2323	<0.0001
C18:3 n-3	0.82	0.84	0.61	0.54	0.6939	<0.0001	0.0738	0.0752	<0.0001
C20:0	0.26	0.23	0.25	0.23	0.1484	0.2461	0.1020	0.0308	0.1671
C20:1 n-9	1.12	0.92	1.25	1.14	0.0273	0.0007	0.1973	0.1610	0.0067
C20:2 n-6	0.72	0.68	0.57	0.52	0.1512	0.0029	0.1375	0.0612	<0.0001
C20:3 n-6	0.08	0.06	0.07	0.05	0.2566	0.6263	0.3749	0.0032	0.4393
C20:3 n-3	0.10	0.07	0.07	0.07	0.0613	0.9618	0.8541	0.0221	0.1175
C20:4 n-6	0.24	0.25	0.14	0.14	0.3164	<0.0001	0.9017	0.0429	<0.0001
PUFA n-3	0.92	0.92	0.68	0.61	0.9053	<0.0001	0.0844	0.0800	<0.0001
PUFA n-6	13.98	14.66	10.28	9.75	0.3307	<0.0001	0.4385	1.2965	<0.0001
PUFA n-6/n-3	15.26	15.90	15.05	16.03	0.0618	0.0168	0.0054	0.6193	0.0133

MH = modern hybrid pigs fed high protein diet; MM = modern hybrid pigs fed medium protein diet; KM = Krškopolje pigs fed medium protein diet; KL = Krškopolje pigs fed low protein diet; RMSE = root mean square error of the model; PUFA = polyunsaturated fatty acids. Significance: NS = p > 0.10, † = p < 0.10, * = p < 0.05, ** = p < 0.01, *** = p < 0.001.
